# The Influence of the Dilution Rate on the Aggressiveness of Inocula and the Expression of Resistance against *Fusarium* Head Blight in Wheat

**DOI:** 10.3390/plants9080943

**Published:** 2020-07-25

**Authors:** Beata Toth, Andrea Gyorgy, Monika Varga, Akos Mesterhazy

**Affiliations:** 1 NAIK Department of Field Crops Research, 6726 Szeged, Hungary; beata.toth@gabonakutato.hu (B.T.); gyorgyandrea88@gmail.com (A.G.); varga.j.monika@gmail.com (M.V.); 2 Cereal Research Non-Profit Ltd., 6726 Szeged, Hungary; 3 Department of Microbiology, University of Szeged, 6726 Szeged, Hungary

**Keywords:** *Fusarium* head blight, resistance, artificial inoculation, deoxynivalenol (DON), *Fusarium* damaged kernels (FDK), disease index (DI)

## Abstract

In previous research, conidium concentrations varying between 10,000 and 1,000,000/mL have not been related to any aggressiveness test. Therefore, two *Fusarium graminearum* and two *Fusarium culmorum* isolates were tested in the field on seven genotypes highly differing in resistance at no dilution, and 1:1, 1:2, 1:4, 1:8, and 1:16 dilutions in two years (2013 and 2014). The isolates showed different aggressiveness, which changed significantly at different dilution rates for disease index (DI), *Fusarium*-damaged kernels (FDK), and deoxynivalenol (DON). The traits also had diverging responses to the infection. The effect of the dilution could not be forecasted. The genotype ranks also varied. Dilution seldomly increased aggressiveness, but often lower aggressiveness occurred at high variation. The maximum and minimum values varied between 15% and 40% for traits and dilutions. The reductions between the non-diluted and diluted values (total means) for DI ranged from 6% and 33%, for FDK 8.3–37.7%, and for DON 5.8–44.8%. The most sensitive and most important trait was DON. The introduction of the aggressiveness test provides improved regulation compared to the uncontrolled manipulation of the conidium concentration. The use of more isolates significantly increases the credibility of phenotyping in genetic and cultivar registration studies.

## 1. Introduction

Breeding for Fusarium head blight (FHB) resistance is the most effective means of providing useful protection of wheat [1,2]. It is well known that resistance to *Fusarium graminearum* and *Fusarium culmorum* is not race-specific [3,4,5,6,7]. Research has shown that in wheat, the resistance to different *Fusaria* is also species-non-specific, e.g., the genotypes, being resistant to *F. graminearum*, will also show resistance to other *Fusarium* spp. tested [8,9]. As pathogenicity is a characteristic of the species, the differences in the disease-causing capacity of the individual isolates for non-specialized pathogens, such as *Fusarium*, is referred to as aggressiveness [10,11,12]. This relates to the primary symptoms; reactions to other traits (*Fusarium*-damaged kernels (FDK) and deoxynivalenol (DON)) might differ. However, their aggressiveness shows a very wide variation [13,14]. “Adjusting” is the process in which a given inoculum will be positioned to a given conidium or fungal mass concentration by dilution or concentration. In this study, the only dilution was tested, and only from previous research that used single isolates. However, it became apparent that resistance expression and aggressiveness are interdependent; at lower aggressiveness, the differentiation of the genotypes for resistance is problematic. Variable aggressiveness in different isolates or in different inocula of the same isolate might influence phenotyping [15,16,17]. To the extent that it is possible, this should be considered in resistance screening, quantitative trait loci (QTL) analysis, and any task that needs reliable and exact data.

The regulation of aggressiveness is a longstanding unanswered question. The early findings were summarized by Dill-Macky [4]. *F. graminearum* is not a good conidium producer, and most isolates are poor in terms of Potato dextrose agar (PDA) [18]. Eide [19] was among the first who performed pathogenicity tests of *F. graminearum* isolates, finding significant differences between them. Snijders and Perkowski [20] (1990) found highly significant isolate aggressiveness differences. However, although the two isolates showed acceptable DI differences, the DON data were inconclusive. A clear differentiation was found in both traits in the case of the IPO-39-01 isolate. Gilbert et al. [10] found similarly large differences in aggressiveness, but here DON was also measured with variable results. Jardine and Leslie [11] found for *F. verticillioides* many-fold differences in levels of aggressiveness at the same conidium concentration of the same isolates. This earlier study is important because it proves clearly that the same conidium concentration does not secure the same aggressiveness in the same genotypes tested. Wu et al. [21] compared aggressiveness in seedlings and heads. In nearly all cases they found significant and positive correlations between seedling and FHB visual scores at a mean r value of about r = 0.50. Similar results were published by Mesterhazy [18]. These results raise the idea that a seedling test could be useful for preselecting of the inocula for field tests. 

In considering what should be regulated, in most cases, the conidium concentration is the subject. However, Takegami and Sasai [22] found that mycelia are as effective as infectious agents as conidia. Sutton [23] considered all spores and mycelium fragments as valid infecting agents. This was also proven for ascospores and macroconidia were considered as generally equivalent infecting agents [24,25]. Grausgruber et al. [26] found that all propagula could infect, and therefore, they spoke about colony-forming units (CFU). Finally, the possibility of directly regulating aggressiveness has been suggested [13,27]. In the dilution test [27], an isolate was found where 20-fold dilution in the seedling test did not change aggressiveness. However, in another isolate, the aggressiveness was reduced to near zero at the same dilution rate. As there were no field experiments at hand, in the current study, it was decided to investigate this problem in field inoculation tests. 

No clear tendency has been found in aggressiveness related to the media used for inoculum production [4]. The correlation between conidium concentration and aggressiveness has rarely been studied. Stein et al. [28] tested inoculum concentration treatments, including suspensions of 0, 100, 500, 1000, 5000, 10,000, 50,000, and 100,000 conidia/mL in sterile, deionized water (sdH_2_O). For the test, a highly aggressive isolate called Fg4 was used. The correlations between conidium concentration and DON contamination were highly significant for grain (r = 0.93, *p* = 0.001) for both greenhouse and field experiments. However, as only one isolate was tested, general conclusions cannot be made. The correlations with incidence and severity were much looser, in agreement with Mesterházy et al. [16]. Stack and McMullen [29] also found a close correlation until a maximum was reached, and thereafter, no additional change was seen. Because only one inoculum was tested in different dilutions, the functions should not necessarily be the same for other tests.

In the current study, a number of papers were screened (Table 1) regarding the applied methodology [3,20,21,26,30,31,32,33,34,35,36,37,38,39,40,41,42,43,44,45,46,47,48,49,50,51,52,53,54,55,56,57,58,59,60,61]. The cited papers, relating mostly to wheat and published during the past several decades, give no information regarding the means of adjustment, whether by dilution or concentration. It is possible that the stock suspension may either need dilution to reach the wanted conidium concentration (such as 5 × 10^5^ conidia/mL) or must be concentrated when its density is too low. No details were given in the literature [26,27,28,29,30,31,32,33,34,35,36,37,38,39,40,41,42,43,44,45,46,47,48,49,50,51,52,53,54,55,56,57,58,59,60,61], with the expression “adjusted” used in most cases. It appears that the researchers did not think that this practice could influence experimental results. Moreover, there is a significant variation in the conidium concentrations used (10^4^–10^6^/mL), with 5 × 10^5^ /mL the most commonly used concentration. In one previous paper, different concentrations were used for spray and point inoculation [49]; however, no explanation was provided why the given concentrations were used. The conclusion, therefore, is that there is no agreement regarding which the optimal concentration. None of the reviewed papers mentioned that the regulation of the conidium concentration was originally intended to regulate aggressiveness nor presented any data about the aggressiveness of the given inoculum before inoculation. Thus, it appeared that most authors, first, regulated the conidium concentration because of usage, and second, because they did not consider that this could cause a serious problem. As genetic differences in aggressiveness level between isolates exist, this does not support the existence of a generally used conidium concentration for every isolate. However, many more papers reported on the use of a highly pathogenic isolate based on previous experience. This means that aggressiveness was considered an important factor, but was not connected to the conidium concentration problem.

Most previous studies worked with *F. graminearum*; in several cases in cooler regions, *F. culmorum* was mentioned [26,27,28,29,30,31,32,33,34,35,36,37,38,39,40,41,42,43,44,45,46,47,48,49,50,51,52,53,54,55,56,57,58,59,60,61]. Aggressiveness was evaluated by the FHB visual head data. Eighteen papers used point inoculation (P) and 24 presented spraying inoculation (S) results. Several papers dealt with the fungal genetics problem when the inoculum was required, but plant infection was not conducted. Two main inoculation methods were used: Spraying inoculation, in which heads were sprayed by a conidium suspension, or point inoculation, in which one floret in the middle of the head was inoculated. Therefore, adjustment of the conidium concentration (or fungal mass concentration) is a general problem for all inoculation methods. The proportion of studies we identified that used point inoculation was higher (19 P *versus* 24 S) than its proportion in the review paper in Buerstmayr et al. [62] who identified more than 60 QTL by P and more than 110 by S inoculation. Of the 44 cases in this previous study, 7 cases reported very high aggressiveness, 14 good, 7 medium, 6 low, and 9 cases were not documented or mentioned. Zwart et al. [63] spoke first about overall resistance to FHB by combining Type I and Type II resistance together, their inoculum had 1 × 10^5^ cinidia/mL. We also detected significant variability in aggressiveness at the same conidium concentration. It should also be considered that not all experiments could be published because the aggressiveness was not high enough to demonstrate convincing genotype differences. Therefore, it is supposed that the problem is larger than the analysis of the published data shows. All reviewed papers [26,27,28,29,30,31,32,33,34,35,36,37,38,39,40,41,42,43,44,45,46,47,48,49,50,51,52,53,54,55,56,57,58,59,60,61] stress the significance of the toxin problem, but toxin data were published in only 15 cases, and no data were given in 29 cases. From these 15 cases, values in 6 were too low to detect reliable differences in response. Seven papers reported high or very high DON concentrations and two produced medium level DON. Therefore, the question arises, how can genotypes be selected for low toxin contamination, when toxins are not tested? Therefore, resistance expression does not only involve checking for visual symptoms, but also testing for FDK and DON contamination.

In recent decades, important research has been conducted in Szeged, Hungary, using four or eight isolates independently [16,17,64]. In all cases, the response to FDK and DON was regularly tested, providing the motivation to extend these previous studies to consider the dilution problem at different resistance levels using four isolates, and also to investigate the FDK and DON response under different dilution rates. This is an unusually extensive and complex approach to the problem to better understand the processes involved in forming disease response and the significance of the dilution in this process. 

The main objectives of this study were to examine: (i) The aggressiveness response of the isolates at different dilution grades; (ii) the relationship of visual symptoms to FDK and DON; (iii) how the expression of resistance may be influenced by the dilution effect for the three traits; and (iv) how a more precise phenotyping system could be developed.

## 2. Results

### 2.1. Visual Symptoms and Disease Index (DI)

**Figure 1:**
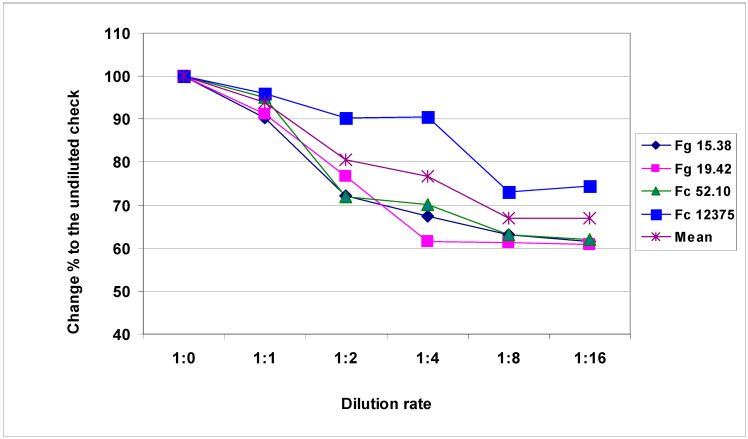
Reduction of the aggressiveness of *Fusarium graminearum* and *Fusarium culmorum* isolates at different dilution rates, visual symptoms, DI%, across genotypes, 100 = undiluted inoculum, 2013-2014.

Highly significant differences (about three-fold, considered the means) in aggressiveness were found for the isolates investigated, as shown in Table 2. It is also clear that the dilution reduced aggressiveness by about one-third, as indicated by the comparison of the aggressiveness of the original inocula and the 1:16 dilution. The correlations between the dilution responses for the three lower aggressive isolates were very close, but the correlations between these responses and the most aggressive Fc 12375 were more variable. To compare the influence of the dilutions, we set the non-diluted inoculum to 100%, and thereafter, the reduction was measured for all isolates along with the dilution grade (Figure 1). It appeared that the reduction was higher in the case of the less aggressive isolates, even though they differed nearly two-fold in aggressiveness. The difference was largest between isolates at 1:4 dilution. The reduction varied between 90% and 60%. Table 3 presents the DI means for isolates in the three experiments reflecting the aggressiveness differences. The independent inocula from the same isolate showed very close results in 2013, indicating that their aggressiveness was very similar although their biomass content might be different. 

**Figure 2:**
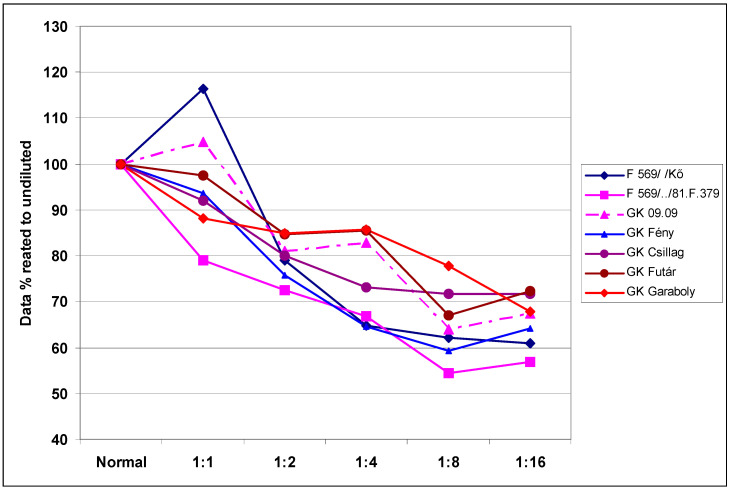
Evaluation of disease response of winter wheat genotypes to dilution at different dilution rates related to the non-diluted original inoculum (100%), visual symptoms DI%, across isolates, 2013–2014.

**Figure 3:**
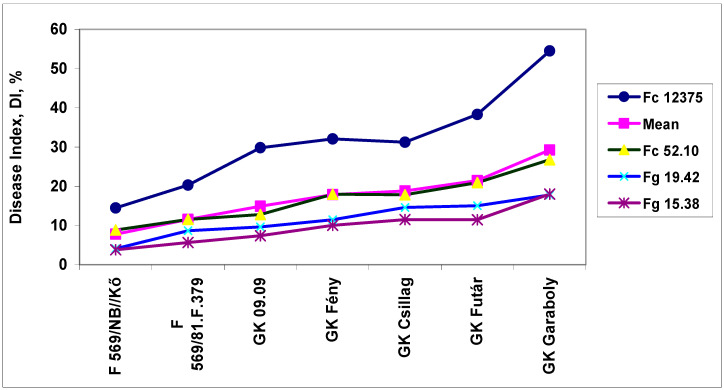
The resistance of winter wheat genotypes to FHB. DI of the seven cultivars at the different isolates, Szeged, 2013 and 2014. LSD 5% between any data points except mean—1.92; for the mean data—0.96.

The genotypes also reacted differently to the dilution rates (Table 4). The resistance difference between genotypes was larger than three-fold for the means across dilutions, but different at different dilution rates. The mean dilution responses were very similar for DI data, as presented in Table 2. It appeared that the least dilution (1:1, 6.0% decrease) did not drastically reduce the aggressiveness. It is probable that such a dilution is tolerable when there is a shortage of inoculum, for example. Higher dilutions caused a significantly higher reduction in aggressiveness; at 1:16 dilution, there was a 33% mean reduction across genotypes. Therefore, the aggressiveness resulting from a higher dilution rate can be unpredictable. The cultivar differences were four-fold, and a decreasing trend following increasing dilutions was found in all cultivars. To separate the data from the resistance level, we set the performance of the non-diluted response to 100%. The dilution rate caused the largest difference at a 1:1 ratio, which yielded responses of between 79% and 116% (Figure 2), with a difference between maximum and minimum values of between 12% and 20%. Overall, this data clearly shows that there is an interaction between the resistance level and the dilution rate. However, this does not explain why the most susceptible cultivars, GK Futár and GK Garaboly, behave differently. An increase was recorded at a 1:1 dilution for the two genotypes, but these were not among the most susceptible. For the other genotypes, only a reduction was detected. It appears that other effects were also influencing the reactions. The reactions of the genotypes to the different isolates exhibited parallel running curves: The less aggressive isolates showed less convincing genotype differences, but the most aggressive isolates produced a much better differentiation of the genotypes (Figure 3). 

### 2.2. Fusarium Damaged Kernels (FDK)

**Figure 4:**
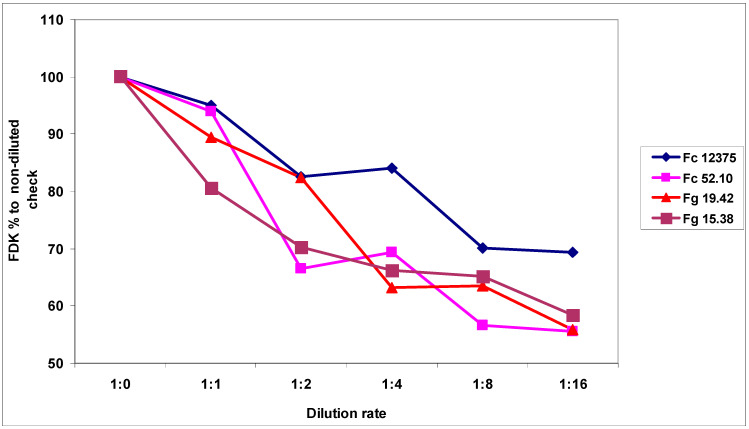
Reduction of the aggressiveness of *F. graminearum* and *F. culmorum* isolated at different dilution rates, FDK data, 100 = non-diluted inocula across genotypes, 100 = undiluted inoculum, used as check, 2013 and 2014.

The magnitude of the reduction following dilution was about one-third (Table 5), similar to the case of the visual symptoms caused by the different isolates. The decrease across isolates was significant, with the exception of the difference between the 1:8 and 1:16 dilutions. For aggressiveness, all isolates differed significantly from the others. The aggressiveness of the non-diluted inoculum decreased by 30.6%, and for others varied between 41.5% and 44.4%. This means that the risk of a large reduction in aggressiveness is lower for highly aggressive isolates. Nonetheless, a similar pattern can be seen in the correlation between isolates and the dilution rates (i.e., r values between 0.90 and 0.97, and significance at *p* = 0.02 or *p* = 0.01). The relative decrease to the non-diluted control indicates that a decrease of FDK appeared variable for the four isolates (Figure 4). All dilutions reduced aggressiveness to a different degree. The most aggressive isolate showed a significantly smaller decrease than the other less aggressive isolates. The highest FDK was found in 2013 at 33.92%; in 2014, the two tests showed kernel infection rates of 13.52% and 15.15% across all genotypes. The correlation between 2013/2014A was r = 0.89 (*p* = 0.02), 2013/2014B r = 0.63 (ns; not significant), and 2014A/2014B showed r = 0.56 (not significant).

**Figure 5:**
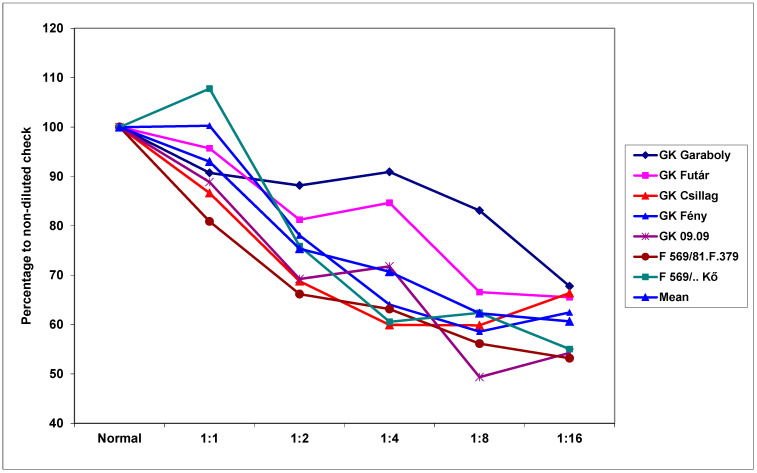
Reduction of the aggressiveness of *F. graminearum* and *F. culmorum* isolates at different dilution rates, FDK data, 100 = non-diluted inocula across isolates, 2013 and 2014.

**Figure 6:**
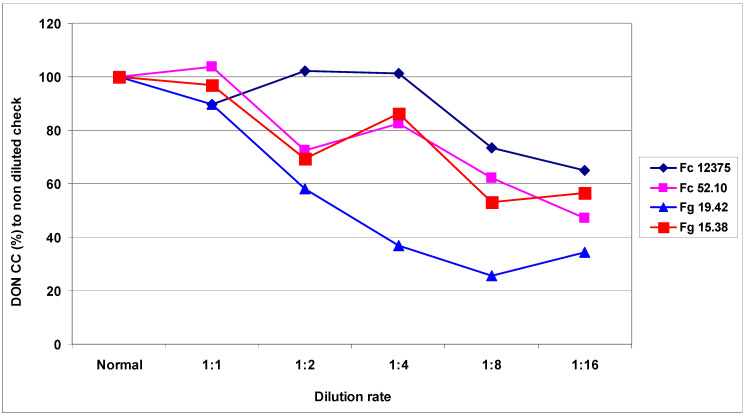
Reduction of the aggressiveness of *F. graminearum* and *F. culmorum* isolated at different dilution rates, DON data related to the non-diluted original inoculum, 2013 and 2014.

The cultivars showed a greater reaction difference to the dilution rate measured by FDK (Table 6A). The resistance differences increased to more than 5-fold compared to the 3.5-fold increase of DI. No significant difference was found between GK Csillag, GK Fény, and GK 09.09. However, the difference between the most and second-most susceptible genotype was much larger than was seen for the visual symptoms. The correlations between cultivar responses to dilutions (*n* = 7) showed a variable picture (Table 6B). GK Garaboly presented significant correlations with the two other cultivars. The others showed a significant relationship at different significance levels. The mean responses to the dilution rates differed significantly. However, no significant difference was found between means of 1:2 and 1:4 dilutions, or 1:8 and 1:16 dilutions. The data for the relative FDK values at different dilution levels compared to the non-diluted original concentrations are shown in Figure 5. The smallest difference was found between the non-diluted and 1:1 dilution rate. When the quantity of suspension was less than required, a dilution up to this rate did not cause a significant change in aggressiveness. There was a clear distinction between the susceptible cultivars and those that are more resistant, namely, the susceptible cultivars showed a smaller decrease at different dilution rates (Figure 6). The most resistant genotype showed an increase at 1:1 dilution; thereafter, however, a rapid decrease was registered. It appears that the effect of the dilution depends both on aggressiveness and genotype resistance. At a dilution of 1:1, the data varied between 80% and 108%, at 1:4 between 60% and 90%, and at 1:16 the two previously mentioned groups diverged significantly, and the difference was smaller (approximately 10%).

### 2.3. DON Evaluation

DON contamination resulted in significantly larger differences than shown in the DI of FDK. The mean for Fc 12375 was 22.40 mg/kg, and the value for the least aggressive Fg 15.38 was 3.36 mg/kg (Table 7A). The reduction of DON was different: The most aggressive isolates suffered a 35% reduction, and the less aggressive isolates showed reductions of 47%, 34.5%, and 57%; these values were significantly higher than those of DI and FDK. However, Fc 12375 did not suffer a significant loss in aggressiveness for dilutions up to 1:4 dilutions. For the other dilutions, a reduction was seen much earlier. The correlations between isolates and the different dilution grades varied (Table 7B). The higher values for Fc 12375 did not significantly correlate with the data of the other isolates. The relative DON data showed high variability in isolate response to dilution. Figure 6 presents the decrease of the DON contamination at different dilution stages, in which the most aggressive isolate followed the pattern of DI and FDK. The isolate Fc 12375 did not significantly correlate with any of the data of the other less aggressive isolates. However, the correlations between the less aggressive isolates were all significant. Additionally, the three least aggressive isolates were divided: Two isolates were close to each other, but isolate Fg 19.42 followed a different function. It appeared that the 1:1 dilution did not greatly influence the aggressiveness measured by DON contamination. Thus, doubling the dilution (i.e., 1:1) was tolerable when an insufficient amount of inoculum was produced (Figure 6). The 1:1 dilution had a smaller effect, and on average showed a non-significant influence on DON production, with greater differentiation seen afterwards. The Fc 52.10 and Fg 15.38 isolates produced very similar responses to dilution, despite three-fold differences in DON contamination. This indicates that the DON data cannot be extrapolated from the DI or FDK data.

**Figure 7:**
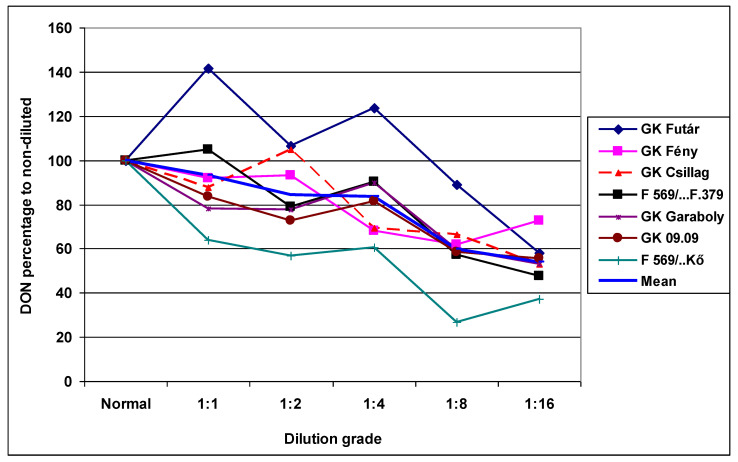
Reduction of the DON contamination at genotypes with differing resistance and at different dilution rates, relative DON data as % to the non-diluted control inoculum, 2013 and 2014.

The differences between cultivars were also more clearly expressed (Table 8A). The resistance differences were highly significant, and a five-fold difference was seen between the most resistant and most susceptible genotypes. The resistance ranking differed for the different dilutions, indicating that this problem may be seen in regular experimental practice. The difference between dilution grades was significantly smaller, between 12.56 and 6.94 mg/kg (Table 8A). The smallest decrease, of 35%, was found in the most aggressive Fc 12375 isolate; the three other isolates showed decreases of 52%, 65%, and 43%. These decreases were larger than those found for DI and FDK. These deviations are shown in Figure 7, where the performance of the given cultivar is shown as a percentage of the non-diluted original inoculum. When dilution did not have the same influence on aggressiveness for the isolates used, nearly parallel curves with stable resistance differences can be seen. This is, however, not the case. It is not possible to forecast the effect of the dilution. Interestingly, the genotype reactions differed at different dilution grades. The correlations were significant in 11 cases of the 21, and only two cases showed significance at *p* = 0.001 (Table 8B). However, in less aggressive isolates, the cultivar differences were not as significant as in the most aggressive Fc12375 isolate. At a high level of aggressiveness, there was a higher probability of finding highly significant differences between genotypes.

Between experiments, DON presented the largest differences: The 2013 value was 23.3 mg/kg, 2014A was 3.57 mg/kg, and 2014B was 3.55 mg/kg. These differences were much larger than that seen for DI and FDK. The correlations of the experiments varied–2013/2014A was r = 0.89, *p* = 0.02; 2013/2014B and 2014A/2014B realized r = 0.40 and r = 0.47, respectively, without significance. 

The resistance responses of the genotypes for the isolates were not determined for DI, FDK, and DON, because the main task of the study was to test the dilution effect. However, in previous studies, only one isolate was used. For this reason, the means by which different isolates work in a resistance test, and whether isolates show deviations or the same patterns, is significant. The basic table (Table 9A) presents the data that served as the basis of the correlation analyses. The corresponding data were paired for each isolate, and the correlations between traits were the computed (Table 9B,C). All FHB/FDK correlations were significant between *p* = 0.01 and 0.001 for all isolates; the value was lower only for Fg 19.42 (Table 9D). The FHB/DON correlations were lower: Two had significance at *p* = 0.05, Fc 12375 recorded *p* = 0.9078, and one showed no significant relationship. For FDK/DON, correlations were higher for all cases. The lower value for only Fg 19.42 indicates that aggressiveness and DON production does not have a significant relationship. The correlation across isolates allowed a comparison of the individual isolates, which showed Fg 19.42 was poorer than the other isolates. The difference between DI/DON was generally smaller than that of the FDK/DON relationship (Table 9D), but the largest difference was recorded between the performances of the means of the isolates. This indicates that the use of more isolates has a positive effect on the reliability of the results.

ANOVA (Table 10) showed highly significant main effects for all traits tested. The genotype, isolate, or year effects were not surprising; we concentrated on the effect of the dilution (Table 10A). The dilution effect was smaller than the genotype effect for all traits, and the isolates differed greatly in their aggressiveness. The highest middle of squares (MS) values were found in the experiment × isolate interaction, indicating that the aggressiveness of the given isolates was not stable in the experimental series. In this analysis, the absolute numbers were less important, but the change in ranking. In the current study, the two-way interactions containing genotype, dilution, and isolate were more important. The main effects were much larger than the corresponding interactions A × B, A × C, and A × D. A significant difference in the main effect and the interaction means that the genotype effect is relatively stable at different levels of the other variable (Table 10B). For this reason, the main effects were tested against the corresponding interactions. In all traits, the main effect of the genotype was significantly higher than the A × B interaction, indicating that the resistance level played the decisive role and that dilution was similarly significantly different from that of the A × B interaction. Thus, its significant influence was also supported in this respect. The genotype × isolate interaction was much stronger, and the resistance ranking played a more significant role (e.g., the higher A × C interaction), even though the genotype effect was significantly superior to the A × C interaction. This was also true for the isolate main effect. The isolate × dilution interaction was small, even though it was significant, and both the isolate and dilution main effects significantly differed from the interaction, e.g., their dominant role against the interaction was clearly proven.

### 2.4. Comparison of Traits

**Figure 8:**
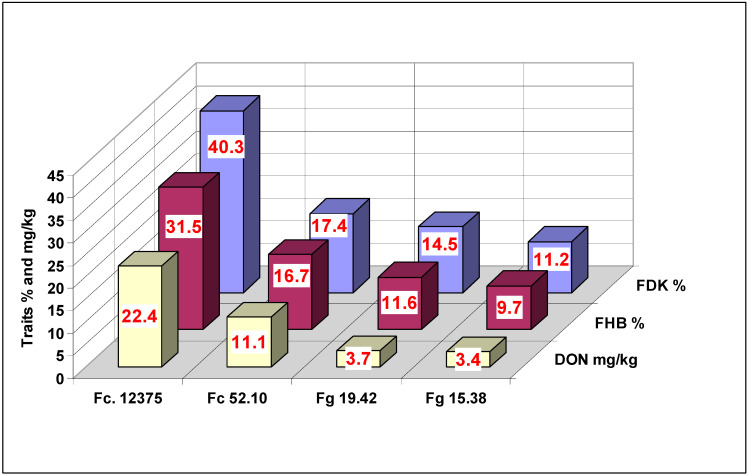
Performance of the isolates tested for the three traits in the inoculum dilution test against FHB in wheat, 2013 and 2014. LSD 5%: DI = 0.72, FDK = 1.53, DON = 0.99.

The general means of the three traits across dilution rates and genotypes (Figure 8) indicated the different characteristics of the given isolates. The two *F. graminearum* isolates were significantly lower DON producers; only 25–29% of the FDK value could be achieved. The different *F. culmorum* isolates had corresponding values of 55% and 63%. This does not mean that the *F. culmorum* isolates would be generally better DON producers; the only point is that the different DON-producing isolates follow different patterns regarding not only FHB and FDK values, but also for DON. As correlations between traits for the different isolates are between 0.967 (*p* = 0.05) and 0.991 and 0.991 (*p* = 0.02), the reactions can be shown to be similar but not identical. 

**Figure 9:**
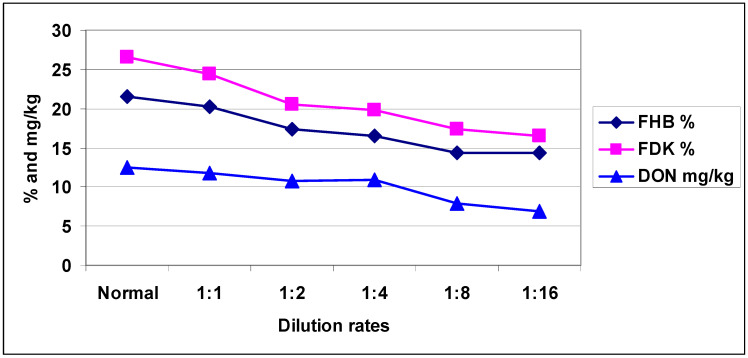
Average aggressiveness of the isolates used for FHB %, FDK % and DON contamination (mg/kg) at different dilution rates across isolates and genotypes, 2013 and 2014. LSD 5% FHB (DI) = 0.89, FDK = 1.41, DON = 1.21.

The mean of the four isolates (Figure 9) shows that the responses were much more similar than they were against the individual isolates. There were visible differences, but the correlations were between traits at different dilution rates (r = 0.92–0.99). There were 252 replications for each data point, and the correlations thus have a solid basis. We should remark that the DI and FDK did not change between the last two dilutions, but DON showed a further decrease at 1:8 and 1:16 dilutions. 

**Figure 10:**
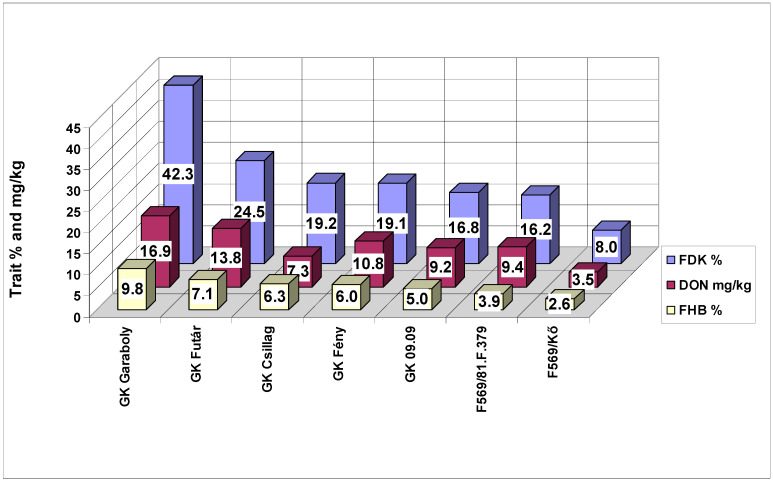
Performance of the genotypes tested for the three traits in the inoculum dilution test against FHB in wheat, 2013 and 2014. LSD 5% FHB (DI) = 0.96, FDK = 1.52, DON = 1.31.

The cultivars used showed similar performances to the different traits (Figure 10). The one exception was GK Csillag, which produced the second-lowest DON value, and it was less than those of the other three genotypes that had lower values for FHB and FDK. In another experimental series, this cultivar showed the same result (Mesterhazy, unpublished), suggesting this is a significant phenomenon. The correlations between traits were somewhat lower (FHB–DON r = 0.9558 (*p* = 0.001) and r = 0.8912 and 0.9155 (*p* = 0.01). For this reason, correlations themselves are not sufficient to infer the relevance of these traits, and the data points should also be checked.

## 3. Discussion

### 3.1. Aggressiveness and Dilution Rate

The lesson from the previous papers reviewed [30,31,32,33,34,35,36,37,38,39,40,41,42,43,44,45,46,47,48,49,50,51,52,53,54,55,56,57,58,59,60,61] shows clearly that the present practice of regulating conidium concentration has severe problems. It appears that the authors of this previous research fulfilled a formal requirement to apply a given or expected conidium concentration. However, no aggressiveness tests were made to examine the process of inoculum production. Furthermore, previous studies did not indicate that a specific conidium concentration would be required to appropriately regulate aggressiveness [30,31,32,33,34,35,36,37,38,39,40,41,42,43,44,45,46,47,48,49,50,51,52,53,54,55,56,57,58,59,60,61]. Some authors mentioned that the isolate that was applied was aggressive based on the experience of past tests. The traits measuring aggressiveness (e.g., symptom severity) were varied: Eleven cases used the disease index, and others examined incidence or severity, typically 21 days after inoculation or AUDPC, when more ratings were made. Evaluation was conducted based on different scales, either in percentage terms or using the traditional 1–9 scale used from breeding practice. All of the previous studies we reviewed described only a part of the FHB resistance. A more significant problem is that a large number of the sources (15) recorded only medium or low infection data, indicating that the formally used adjustment of conidium concentration is not an effective means regulating aggressiveness. In the tests conducted in 24 studies, no FDK and DON data were published, even though the significance of toxin contamination was heavily stressed in each paper. From the 15 papers in which DON was measured, only seven produced acceptable DON levels. This means that only half of the papers were in a suitable position to evaluate genotypes or other differences in the tests—related to the 44 papers, this is only 15%.

The results in the current study show that the relationship between dilution rate and aggressiveness is complicated. Comparing results in a previous study [13] with those found here, the more aggressive isolates were more stable in both tests. The baseline aggressiveness was very similar for the five isolates tested in 1977. Nonetheless, the most aggressive isolate showed an increase at 20-fold dilution, and the least aggressive caused no damage at a dilution ratio of 1:20. It was also similar in that the response to the dilution cannot be predicted exactly. In the present FHB test, the variation was relatively high. The aggressiveness of the most aggressive isolate was reduced only moderately, but the remaining three isolates, which had significant differences, responded similarly to the dilution. This indicates that a given conidium concentration may lead to different disease severities, as suggested in the cited literature. 

The impression given by the earlier research is that aggressiveness is thought to be stable. It is known [65] that *Fusarium* isolates normally lose aggressiveness, implying that a change is necessary at some stage. A passage may help, but in most cases, more aggressive new isolates should be involved in the tests. There might be also other causes. In seedling tests, the aggressiveness of the isolates varies [66]. Under field conditions, the visual symptoms, FDK, or DON can vary strongly because temperature, humidity, rain, and other conditions are not stable [5,7,64]. Therefore, the differences between years are mostly due to these events. In the case of only one inoculum, the stability cannot be tested, and the variation in response will be understood as a year effect. Following a previous study [13], the Szeged tests were conducted using four different isolates [5,7,9,12,64], in which highly significant isolate × year interactions were found. Another experience [2] showed that parallel inocula from the same test tube at the same time showed variable aggressiveness. Therefore, the probability is high that a given conidium concentration will result in different aggressiveness levels. 

The previous research has identified a number of pathogenicity genes that were expressed very differently in different isolates [66,67,68,69]. The probability is very low that all of these genes correlate with aggressiveness [20]. It is more probable that each *Fusarium* isolate has a different mix (with unknown numbers and functions) of these genes. Therefore, the conidium concentration is only one of the traits that influence aggressiveness. For this reason, the belief that a given conidium concentration can reach the target to secure the wanted aggressiveness level is a false assumption, which is clearly shown by the cited literature [30,31,32,33,34,35,36,37,38,39,40,41,42,43,44,45,46,47,48,49,50,51,52,53,54,55,56,57,58,59,60,61]. 

The comparison between field and laboratory aggressiveness tests [18,70] showed that the same inocula of the same isolate resulted in a good correlation. Since we did not regulate conidium concentration, we directly tested aggressiveness [13,27]. Wu et al. [21] also found medium level correlations between seedling and FHB tests. These make it possible to screen inocula at the seedling stage, which is of benefit in FHB resistance tests. 

The experience of several decades shows that the aggressiveness test will not alone resolve problems relating to resistance testing. It is clear that the low- and non-aggressive inocula can be discarded easily. However, one–two isolates have occasionally produced poor field results. This means that, although a previous aggressiveness test may serve better than an arbitrary conidium or fungal biomass concentration, it does not secure high aggressiveness for the experiments. 

The aggressiveness test (Figure S1, Table S1) shows the dilutability of the given inoculum. Better field performance is achieved with an inoculum where all ratings are zero, such as Fc 12375, e.g., during the evaluation, no healthy germ could be identified. Among inocula that have all zero at the original concentration and higher numbers at 1:1, 1:2, and 1:4 concentrations, disease severity in the field is lower. To secure high aggressiveness, typically dilution is not used, but when there is a shortage in inoculum, a dilution of 1:0.5 or 1:1 is suitable. The results shown here indicate that this dilution rate is unlikely to result in significant changes in aggressiveness [71]. This also means that so much inoculum should be produced that under normal conditions would be enough for the whole experiment without changing inoculum. 

The data clearly show that forecasting aggressiveness is not possible based on the conidium concentration and that the risk is greater when the level of FDK of DON level should be forecasted. In addition to conidium concentration, quantitative PCR is suitable for the estimation of fungal mass (Gosman et al. [61]). The correlation between the fungal mass in the grain of different genotypes and DON contamination was r = 0.61. Furthermore, the number of colony-forming units can be used as the basis for regulation [26]. However, without an aggressiveness test, they provide no advantage compared to the conidium concentration. It is well known that the production of DON is a virulence factor of the fungus. Therefore, selection for high aggressiveness is generally accompanied by a high DON production ability. Similarly, previous experience also has a major significance in choosing the right isolate.

In summary, dilution significantly changes the aggressiveness of the given inoculum, especially at higher rates. As shown in the cited literature, without information about the original concentration, the applied dilution rate cannot be known. Based on the largest and smallest conidium concentrations (10^4^ and 10^6^ /mL, respectively), the possible maximum dilution rate can reach 1:100. Because the dilution rate normally decreases the aggressiveness of the inoculum, it automatically decreases resistance differences, even if high aggressiveness is the focus. It is suggested that a direct aggressiveness test can better serve the targets of the experiment and possibly produce high aggressiveness. This is valid mainly for producing inocula for breeding, selection, and phenotyping purposes. In other tests, the conidium concentration will retain its significance. 

### 3.2. Resistance Expression

To the best of our knowledge, the relationship between resistance expression and dilution has not been analyzed. The resistance level is genetically fixed, but the rate of the disease depends on epidemic severity, ecological, and other conditions that influence the build-up of an epidemic. In this process, both dilution and aggressiveness play an important role. Dilution has a direct influence on resistance expression. As for aggressiveness decreases, the probability of highly significant genotype differences is reduced. We found deviations in the response of different cultivars to different dilution rates. The genotype ranking was often different, and in FDK the maximum–minimum differences at a given dilution rate were wider by about 30%, and genotype ranking differed at different dilution rates. This means that dilution can strongly influence the maximum–minimum rates. Thus, the best and worst genotypes were the same, but the difference varied between them. In contrast, genotypes with smaller differences often changed position. Another problem is that one isolate produces only one aggressiveness level; therefore, the degree of resistance cannot be estimated properly; more parallel isolates provide more reliable results. Because *F. graminearum* and *F. culmorum* are highly pathogenic species, the selection of highly aggressive isolates is not a major concern. For less pathogenic species, however, this needs careful work. 

The cultivar GK Csillag is worthy of interest. During the past 12 years, it has survived several epidemics of varying magnitudes. The reaction of the heads was comparable to a more susceptible cultivar, but DON contamination was significantly lower. The data clearly show that it was the second-best genotype in terms of DON; but for FDK and DI, it ranked only in fifth place. This finding was valuable, because for some time Csillag was the most popular cultivar in Hungary. Of the present genotype collection, the resistance level of F569/Kő is higher, but among regular winter wheat cultivars, this seldom occurs. The single yearly inoculation with four isolates did not yield a definitive result and only signalized a tendency. Rather than 4, in this test, we had 4 × 6 = 24 epidemic situations, thus proving the usefulness of additional DON resistance is a reality. This complexity and additional DON resistance were also proven in a previous methodical study [16]. 

### 3.3. Breeding and Scientific Aspects

The tests clearly showed that the regulation of the conidium concentration—or, more specifically, the concentration of the CFU (which, in addition to the conidia, also includes mycelium fragments)—is not equivalent to the regulation of the aggressiveness. Through regulation of the aggressiveness by direct laboratory tests, we increased the probability of securing the desired level of aggressiveness [13,18]. From Takegami and Sasai [22], we know that infection traits (such as mycelium) are equivalent to conidia. This is also valid for the conidia and ascospores [24,25]. 

Breeding programs require two types of inoculation methodology. The first is the production of large quantities of inoculum; 1000 L or more of inoculum should be prepared. Because this cannot be produced with ascospore, the conidia and conidium + mycelium-containing inocula should be chosen. Conidium production requires an additional cleaning phase to separate conidia from mycelium. For large quantities, this is a considerable problem. Nevertheless, using inocula with conidia and mycelium can provide a simple solution for this problem. The aggressiveness test provides direct information on the usefulness of the given inoculum to secure the infection pressure needed. Thus, scientific control can help the control of the inoculum production in the necessary quality. Because the single inoculum of the present methodology is not sufficiently reliable, even aggressiveness tests are made, this does not solve the problems coming from the use of single isolates. This is the reason of the suggestion to use parallel more isoltes. The testing of parental lines requires similar care. Because FDK and DON responses often differ from that of the disease index, their control is highly important in advanced stages of breeding. For mass selection, an aggressive isolate or a mixture of isolates is sufficient.

## 4. Materials and Methods 

### 4.1. Plant Material

Seven winter wheat genotypes were tested with differing resistance levels (Table 11). Four registered cultivars were chosen from the Szeged pool, and three lines from the FHB breeding program managed by our Resistance Department were selected.

### 4.2. Field Conditions

**Figure 11:**
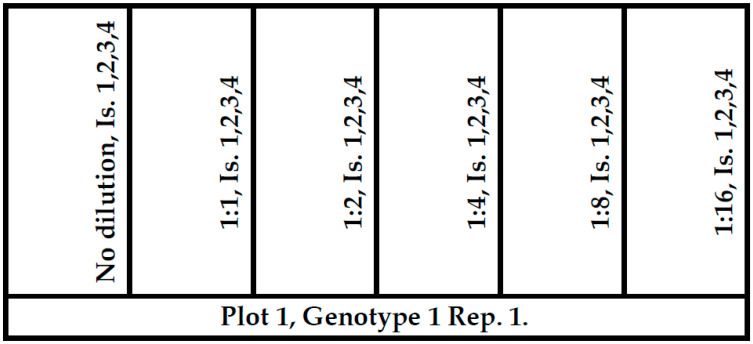
Map of a cultivar plot with six double rows and inoculation plan. The test in a year had 21 such plots. Dilutions: No, 1:1, 1:2, 1:4, 1:8, 1:16. Check = no dilutions, 1,2,3,4. Is = in the figure: Isolates used on groups of heads 1–4, 40–50 cm distance from each other.

One and two experiments were performed in 2013 and 2014, respectively; in total, three independent biological replicates were made. The plant material was evaluated in the nursery of the Cereal Research Nonprofit Ltd. in Szeged, Hungary (46°14′24″ N, 20°5′39″ E) over two seasons (2012/2013 and 2013/2014). The field experiments were conducted in three replicates in a randomized complete block design. Each genotype per replicate was shown in six (1 plot per inoculation date) two-row plots of 1.5 m length in mid-October, using Wintersteiger Plot Spider planter (Wintersteiger GmbH, Ried, Austria). The width of the plots was approximately 40 cm. One experiment in 2013 and two experiments in 2014 were sown for the six dilution rates (1:0, 1:1, 1:2, 1:4, 1:8, and 1:16) (Figure 11). In every two rows, subplots were used for the four isolates at about 50 cm distance within the plot. Therefore, a plot contained 24 inoculations. In the test, 21 such plots were made, and randomization was made according to the genotypes. This resulted in 1512 inoculations for the three experiments, and the same number of FDK estimation and DON analyses. The two experiments were carried out in 2014 with different inocula of the same isolates. Thus, the three tests were able to be analyzed in one ANOVA test. 

### 4.3. Inoculum Production and Inoculation

As in the earlier experiments [6,16], four independent isolates were used. In this test series, two *F. graminearum* (Fg 15.38, Fg 19.42) and two *F. culmorum* (Fc 52.10 and Fc 12375) strains were used, all isolated from naturally contaminated wheat grains, with the exception of Fc 12375, which was isolated from a wheat stalk inside space mycelium from a greenhouse test in 1977. For identification, the Booth manual [65] was used, and the identities of the single spore isolates were confirmed later by *Fusarium*-specific PCR markers [72,73]. The others were younger; 3–4 years ago, three of the older isolates were replaced by more aggressive new isolates. The two tests from 2014 were conducted on two parallel independent inocula from the same isolate. This was important because different inocula from the same isolate may differ largely in aggressiveness [2]. For this reason, 20–30% more inoculum units were produced, so that in the case of low or no aggressiveness, they could be replaced by a more aggressive unit.

**Figure 12:**
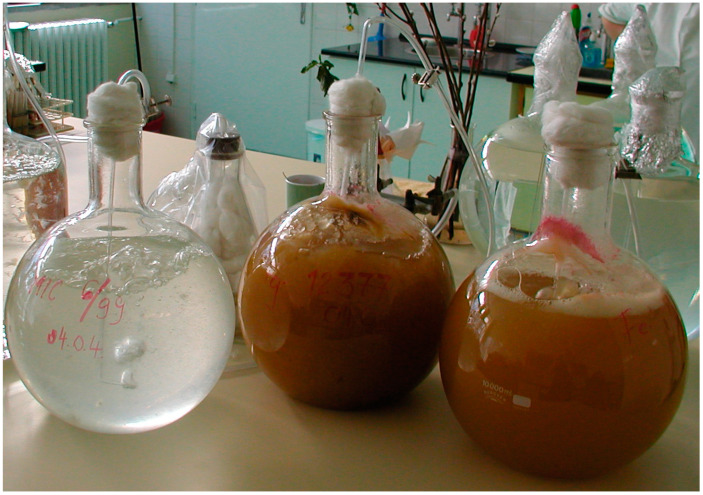
Bubble-breeding (BB) method for inoculum production. Left: A young inoculum 2–3 days old, right: Ready inocula for use before terminating the breeding.

To increase inocula, Czapek–Dox medium (1000 g distilled water, 30 g cane sugar, 1 g monopotassium phosphate, 0.5 g magnesium sulfate, 0.5 g potassium chloride, 0.01 g iron sulfate) was produced in the laboratory. The medium was sterilized and autoclaved in 10 L heat-stable glass balloons for two hours at 120 °C, using the bubble breeding method (Figure 12), in which mycelium and conidia were produced [6,16]. This inoculum contained conidia and mycelium in different ratios. This is not unique in the plant pathology literature [74]. 

The liquid media were increased in a 10 L glass balloon [15]. They were aerated by sterile air for one week at room temperature until the suspensions were ready to use. Conidium concentrations were determined by a blood chamber. Inoculation was performed in the field at the full flowering with spray inoculation with the original concentration and their dilutions. Four isolates were tested within a plot of two rows, each consisting of 15–20 heads with about 50 cm distances between them to prevent cross inoculation between the isolates. Following inoculation, the sprayed heads were covered by polyethylene bags for 48 h [9,16]. After removing the bags, the groups of heads remained loosely bound at half the height of the plants to prevent disturbance of the assimilation of the leaf system.

The aggressiveness of the produced inocula was measured by the Petri dish method [27]. Petri dishes with 12 cm diameter were used. For each isolate, we used two wheat cultivars, one susceptible and one moderately resistant at the seedling stage. When the suspension was ready, after shaking, 50 mL was separated and mixed in a sterilized blender. Four sterile Petri dishes were prepared with sterile double-layer filter paper for a genotype + one non-inoculated control. Nine milliliters of suspension without dilution was placed in the first; 1:1, 1:2, and 1:4 dilutions were made for the next treatments; and the last was the control with sterile distilled water. Before pouring the suspension, 25 healthy grains previously disinfected by 2% NaClO_2_ were counted into each dish superficially. The suspension was uniformly distributed in the Petri dish, and the seeds were ordered in a 5 × 5 binding with germs upwards. Photos of an aggressiveness test (not from this test) and the 2013 aggressiveness test results from this test are shown in Figure S1 and Table S1. The aggressiveness of the isolates was very similar to the results cited from previous experiments with settled conidium concentration [10,11], and the two inocula of the four isolates for experiments 2 and 3 gave a very similar result, thus indicating a good reproducibility (Table 2). The inocula were stored until use at 4 °C. As the number of flowering plots was evaluated on the previous day, only that amount was taken from the 10 L balloon as was necessary for a given day. This decision was made after the aggressiveness test measured by the rate of diseased germs. After finishing the inoculations, another aggressiveness test was performed to check for possible loss of aggressiveness. Normally this was not the case; in tests conducted in past decades, only several cases occurred, and these isolates were excluded from the test. 

### 4.4. Evaluation of the Disease and DON Toxin Content

Visual symptoms were recorded beginning on the 10th day following inoculation on days 14, 18, 22, and 26 [13]. The percentage of diseased spikelets was estimated as the percentage that corresponds to the disease index. At harvest, every group of heads was placed in a different paper bag with a slip containing the label of the string showing the data of the particular group. Following threshing (Seed Boy, Wintersteiger AG., Ried, Austria), a fine cleaning was undertaken using an air separator (Ets Plaut-Aubry, 41290 Conan-Oucques, France). In both cases, air speed was regulated so that shriveled scabby grains were not blown out. Then, visual grain evaluation was conducted where white, whitish, and rose-colored grains were estimated as a percentage compared to the whole grain sample. For toxin analyses, 6 g of the individual samples were separated for milling (Laboratory Mill 3310, Perten Instruments, 126 53 Hägersten, Sweden). For DON analysis, 1 g of maize sample was extracted with 4 mL of acetonitrile/water (84/16, V/V) for 2.5 h with a vertical shaker. After centrifugation (10,000 rpm, 10 min) 2.5 mL of the extract was passed through an activated charcoal/neutral alumina SPE (solid phase extraction) column (Sigma, Al_2_O_3_ and active coal 20:1 ratio were prepared in the laboratory, and 1 g was filled to the 5 ML SPE column) with a flow rate of 1 mL/min. Thereafter, 1.5 mL of the clear extract was transferred to a vial and evaporated to dryness at 40 °C under vacuum. The residue was dissolved in 500 µL of acetonitrile/water (20/80, V/V).

Liquid chromatographic separation and quantification was performed on an Agilent 1260 HPLC system (Agilent Technologies, Santa Clara, California, USA) equipped with a membrane degasser, a binary pump, a standard autosampler, a thermostated column compartment, and a diode array detector. DON was separated on a Zorbax SB-Aq (4.6 × 50 × 3.5 µm) column (Agilent) equipped with a Zorbax SB-Aq guard column (4.6 × 12.5 × 5 µm) thermostated at 40 °C. The mobile phase A was water, and mobile phase B was acetonitrile. The gradient elution was performed as follows: 0 min, 5% B; 5 min, 15% B; 8 min, 15% B; 10 min, 5% B; 12 min, 5% B. The flow rate was set to 1 mL/min. The injection volume was 5 µL. DON was monitored at 219 nm. All chemicals and toxin standards were purchased from Sigma-Aldrich (Budapest, Hungary). 

### 4.5. Statistical Analysis

The visual data for 4–5 readings for a group of heads were averaged and served as inputs for the ANOVA test. From the yield of every group of heads, the FDK and DON determinations were evaluated and served as inputs for the ANOVA test. The four-way ANOVA test was conducted according to Sváb [75] and Weber [76] using the built-in Excel functions. To evaluate the significance of the two-way and three-way interactions, we followed the methodology suggested by Weber [76]. 

## 5. Conclusions

At present, the experimental practice uses widely varying conidium concentrations, with no explanation for the use of a given conidium concentration provided. Because aggressiveness is a readily heritable trait, the same conidium concentration may result in highly different aggressiveness levels. For this reason, the regulation of conidium concentration per se is not sufficient to secure medium or high levels of aggressiveness required in testing. Therefore, the direct measuring of aggressiveness is suggested. Experimental data show that the adjustment of the inoculum—in the present study via different dilution rates—has a varying influence on aggressiveness. Typically, a reduction is a consequence, but this cannot be forecasted exactly. In addition, high aggressiveness of an isolate in a susceptible genotype may result in a low infection severity in a resistant genotype known from regular breeding praxis. FDK and DON response differ from the hypothesized result—therefore, exact forecasting is not possible. For this reason, DON should be measured to ensure that a genotype with an overproduction of DON [16] is not released into commercial production. 

## Figures and Tables

**Table 1:** Experimental data of Fusarium resistance and pathogenicity tests from papers using one single isolate.

Author	Ref. No.	Plant	Inoculation	Fusarium	Con. Conc.	Aggressiveness Visual	FHB Visual	FHB Min–Max %	FDK	DON
Grausgruber et al. 1995	[26]	W	P	gr.+ c.	45–50 × 10^4^, CFU	good	S	35–65	no	no
Evans et al. 2000	[30]	W	P	gr.	2 × 10^5^	no	no	no	no	low
Giancaspro et al. 2016	[31]	W	S	gr.	1.0 × 10^6^	very high	I + S	0–100	no	no
Lionetti et al. 2015	[32]	W	S	gr.	1.0 × 10^5^	high	I + S	5–80	no	no
Chrpova et al. 2012	[33]	W	S	c.	0.8 × 10^7^	high	VSS 1–9	2.33–4.78	high	high
Kalih et al. 2015	[34]	T	S	c.	7 × 10^5^	high	VSS 1–9	8–59	no	no
Miedaner et al. 1996	[35]	R	S	c.	3 × 10^5^	high	VSS 1–9	2.5–4.8	no	no
Bai et al. 1996.	[3]	W	P	gr.	1000/floret	very high	DI	5–100	no	no
Bai et al. 2001 greenh.	[36]	W	P	gr.	1000/floret	low	S, AUDPC	2000.06.11	no	very high
Bai et al. 2001 field	[36]	W	P	gr.	1000/floret	low	S, AUDPC	0.1–0.8	high	high
Kage et al. 2017	[37]	W	P	gr.	1.0 × 10^5^	no	DI	no	no	no
Gilbert et al. 2000	[38]	W	P	gr.	5 × 10^4^	medium	S	6–43	no	no
Gilbert et al. 2000	[38]	W	S	gr.	5 × 10^4^	medium	S	8–72	no	no
Burlakoti et al. 2010	[39]	W	S	gr.	5 × 10^4^	high	S, AUDPC	206–914	low	high
Nopsa 2010	[40]	W	S	gr.	1.0 × 10^5^	high	DI	4–59	low	low
Cai et al. 2016	[41]	W	P	gr.	1.0 × 10^5^	very high	S	2–95	no	no
Soltanlo et al. 2011	[42]	W	S	gr.	1.0 × 10^5^	n.g.	n.g.	n.g.	n.g.	n.g.
Snijders 1990	[43]	W	S	c.	2.5 × 10^5^	high	DI	0–80	no	no
Snijders, Perkowski 1990	[20]	W	S	c.	2.5 × 10^5^	high	DI	1.5–62	no	high
Salameh et al. 2011	[44]	W	S	gr.	2.5 × 10^5^	high	S, AUDPC	n.g.	no	no
Liu et al. 2012	[45]	W	P	gr.	5 × 10^5^	very high	n.g.	n.g.	n.g.	n.g.
Wu et al. 2005	[21]	W	P	gr.	2.5 × 10^5^	high	n.g.	n.g.	n.g.	n.g.
Audenaert et al. 2013	[46]	W	P	gr.	1 × 10^6^	n.g.	no	no	no	medium
Lu et al. 2013	[47]	W	S	c.	1.0 × 10^5^	high	S	1–70	very high	very high
Lu et al. 2013	[47]	W	P	c.	1.0 × 10^5^	medium	S	1–70	n.g.	low
Mendes et al. 2018	[48]	W	S	gr.	1.0 × 10^5^	high	DI	98–100	high	very high
von der Ohe et al.	[49]	W	S	gr.	5 × 10^5^	low	DI	0.7–3.1	no	medium
von der Ohe et al.	[49]	W	S	gr.	5 × 10^4^	low	DI	nd–60	no	low
Zhang et al. 2010	[50]	W	P	gr.	2 × 10^5^	n.g.	DI	n.g.	n.g.	n.g.
Jung et al. 2010	[51]	W	S	gr.	1 × 10^4^	medium	VSS 1–9	0.6–5.2	no	no
Jung et al. 2010	[51]	W	P	gr.	1 × 10^4^	low	S	1.4–3.6	no	no
Zhang et al. 2018	[52]	W	P	gram.	5 × 10^4^	high	S	5–95	no	no
Fu et al. 2012	[53]	W	P	gr.	5 × 10^4^	high	S	5–100	no	no
Srinavasachary et al. 2009	[54]	W	S	c.	1.0 × 10^5^	no	I + S	n.g.	n.g.	n.g.
Srinavasachary et al. 2010	[54]	W	S	gr.	1.0 × 10^5^	no	I + S	n.g.	n.g.	n.g.
Kubo 2009	[55]	W	S	gr.	5 × 10^5^	low	VSS 1–9	3.8–6.4	low	low
Kubo 2009	[55]	W	P	gr.	2 × 10^5^	medium	VSS 1–9	0.67–3.5	no	no
Makandar et al. 2012	[56]	W	P	gr.	3 × 10^4^	high	I + S	30–90	no	low
Boeven et al. 2011	[57]	T	S	c.	7.5 × 10^5^	medium	DI	20– (36–52)	no	no
Ban and Suenaga 2000	[58]	W	S	gr.	n.g.	very high	DI	2.5–100	no	no
Buerstmayr et al. 2002	[59]	W	P	gr.	5 × 10^4^	very high	S	0.4–38	no	no
Buerstmayr et al. 2002	[59]	W	S	c.	5 × 10^4^	very high	S		no	no
Bowden and Leslie 1999.	[60]	W	no	gr.	5 × 10^5^	no	no	no	no	no
Gosman et al. 2007	[61]	W	S	c.	1.0 × 10^5^	high	DI	n.g.	n.g.	n.g.

**General**: n.g. = not given, no = not tested, **Plant**: W = wheat, T = triticale, R = rye, **Inoculation**: S = spray, P = point, Fusarium: gr. = graminearum, c. = culmorum, **Conidium concentration**: CFU = colony-forming unit, **FHB Visual**: I = incidence, S = severity, VSS 1–9 = visual scale 1–9, DI = disease index.

**Table 2:** Reduction of the aggressiveness of *F. graminearum* and *F. culmorum* isolates at different dilutions, visual screening data (Disease Index %) in 2013–2014. (**A**) Isolates, Disease Index %. (**B**) Correlation coefficients.

**(A) Isolates, Disease Index %**					
**Dilution Rate**	**Fg 15.38**	**Fg 19.42**	**Fc 52.10**	**Fc 12375**	**Mean**
Normal	12.79	15.44	21.63	36.10	21.49
1:1	11.55	14.07	20.57	34.61	20.20
1:2	9.24	11.83	15.54	32.59	17.30
1:4	8.63	9.49	15.19	32.64	16.49
1:8	8.08	9.46	13.67	26.37	14.39
1:16	7.86	9.39	13.41	26.89	14.39
Mean	9.69	11.61	16.67	31.53	17.38
LSD 5% isolates					0.72
LSD 5% dilutions					0.89
LSD 5% between any data					2.89
**(B) Correlation coefficients**					
**Correlations**		**Fg 15.38**	**Fg 19.42**	**Fc 52.10**	**Fc. 12375**
Fg 19.42		0.9815 ***			
Fc 52.10		0.9927 ***	0.9652 **		
Fc. 12375		0.8646 *	0.8361 *	0.8757 *	

*** *p* = 0.001, ** *p* = 0.01, * *p* = 0.05. LSD = Limit of significant difference.

**Table 3:** Mean aggressiveness of the *F. graminearum* and *F. culmorum* isolates of the dilution tests in the three tests, 2013–2014, Disease index data % across dilutions and genotypes (No. replicates = 126).

Experiments	Isolates/Disease Index %				Mean
	Fc. 12375	Fc 52.10	Fg 19.42	Fg 13.38	
2013	55.85	39.04	24.50	16.28	33.92
2014/1	18.68	5.61	4.94	6.43	8.92
2014/2	20.06	5.36	5.39	6.37	9.29
Mean	31.53	16.67	11.61	9.69	17.38
LSD 5% Exp.					0.63
LSD 5% Isolate					0.72

**Table 4:** Reduction of the aggressiveness of the winter wheat genotypes at different dilutions acr5oss isolates, visual screening data (Disease Index %) in 2013–2014.

Genotype			Dilutions Grade, DI %				Mean
	Normal	1:1	1:2	1:4	1:8	1:16a	
F 569/Kő	9.67 *a	11.26a	7.65a	6.26a	6.02a	5.88b	7.79a
F 569/81.F.379	16.14b	12.77a	11.70b	10.77b	8.79b	9.17c	11.55b
GK 09.09	17.89b	18.75b	14.49c	14.81c	11.45c	12.05d	14.91c
GK Fény	23.45c	21.96c	17.78d	15.17c	13.89d	15.07e	17.89d
GK Csillag	23.08c	21.22c	18.45d	16.86c	16.55e	16.56e	18.79d
GK Futár	25.39c	24.73d	21.50e	21.70d	16.99e	18.36f	21.45e
GK Garaboly	34.83d	30.70e	29.53f	29.84e	27.06f	23.63g	29.26f
Mean	21.49	20.20	17.30	16.49	14.39	14.39	17.38
LSD 5% genotype							0.96
LSD 5% dilution							0.89
LSD 5% between any data							2.36

* a–f significant difference within column.

**Table 5:** Reduction of the aggressiveness of *F. graminearum* and *F. culmorum* isolates at different dilutions across experiments and genotypes, FDK data expressed as percentage %) in 2013–2014.

Dilution Rate	Isolates, FDK %				Mean
	Fc 12375	Fc 52.10	Fg 19.42	Fg 15.38	
Normal	48.21	23.61	19.20	15.31	26.58
1:1	45.79	22.19	17.18	12.35	24.38
1:2	39.83	15.69	15.82	10.76	20.52
1:4	40.53	16.36	12.15	10.13	19.79
1:8	33.81	13.37	12.20	9.96	17.33
1:16	33.43	13.11	10.74	8.95	16.56
Mean	40.27	17.39	14.55	11.25	20.86
LSD 5% between isolate means					1.15
LSD 5% between dilution means					1.40
LSD 5% between any data in the isolate columns					3.74

**Table 6:** Reduction of the aggressiveness of the winter wheat genotypes at different dilutions across isolates and experiments, FDK data as a percentage in 2013–2014. (**A**) Dilutions, FDK %. (**B**) Correlations between dilution responses of the genotypes.

**(A) Dilutions, FDK %**													
**Genotypes**		**Normal**	**1:1**		**1:2**		**1:4**		**1:8**		**1:16**		**Mean**
GK Garaboly		32.49c*	29.47d		28.65d		29.54d		27.00c		22.01c		28.19d
GK Futár		19.82b	18.97c		16.10c		16.78c		13.19b		12.98b		16.31c
GK Csillag		17.36b	15.04b		11.94b		10.41b		10.39b		11.52b		12.78b
GK Fény		16.49b	16.54b		12.87b		10.56b		9.66b		10.30b		12.73b
GK 09.09		15.51b	13.78b		10.74b		11.13b		7.65a		8.41b		11.20b
F 569/81.F.379		15.48b	12.52b		10.24b		9.77b		8.69a		8.23b		10.82b
F 569/Kő		6.91a	7.45a		5.24a		4.18a		4.31a		3.80a		5.32a
Mean		17.72	16.25		13.68		13.20		11.56		11.04		13.91
LSD 5% genotype													1.54
LSD 5% dilution													1.40
LSD 5% between any data except means—4.95, c* = a–d differing significance between data.													
**(B) Correlations between dilution responses of the genotypes**													
**Genotypes**	**GK Garaboly**			**GK Futár**		**GK Csillag**		**GK Fény**		**GK 09.09**		**F 569/81.F.379**	
GK Futár	0.866 *												
GK Csillag	0.598			0.818 *									
GK Fény	0.672			0.899 **		0.937 ***							
GK 09.09	0.808			0.984 ****		0.893 **		0.926 ***					
F 569/81.F.379	0.814 *			0.921 ***		0.948 ***		0.915 **		0.957 ***			
F 569/Kő	0.692			0.870 *		0.890 **		0.978 ****		0.876 *		0.878 *	

**** *p* = 0.001, *** *p* = 0.01. ** *p* = 0.02, * *p* = 0.05.

**Table 7:** Reduction of the aggressiveness of *F. graminearum* and *F. culmorum* isolates at different dilutions across experiments and genotypes, measured by DON contamination mg/kg in 2013–2014. (**A**) Isolates, DON mg/kg. (**B**) Correlations between responses of isolates to dilution rates.

**(A) Isolates, DON mg/kg**					
**Dilution Grade**	**Fc 12375**	**Fc 52.10**	**Fg 19.42**	**Fg 15.38**	**Mean**
Normal	25.29c	14.19c	6.38b	4.36a	12.56b
1:1	22.68b	14.72c	5.71b	4.22a	11.83b
1:2	25.81c	10.28b	3.72a	3.03a	10.71b
1:4	25.60c	11.72b	2.35a	3.75a	10.86b
1:8	18.59a	8.81a	1.63a	2.31a	7.84a
1:16	16.40a	6.71a	2.20a	2.47a	6.94a
Mean	22.40	11.07	3.67	3.36	10.12
LSD 5% Isolate					0.85
LDS 5% dilution					0.99
LSD 5% between any data in isolate columns 2.62, a–c differing significance between data.					
**(B) Correlations between responses of isolates to dilution rates**					
**Isolates**	**Fc 12375**		**Fc 52.10**	**Fg 19.42**	
Fc 52.10	0.7085				
Fg 19.42	0.5264		0.8478 *		
Fg 15.38	0.7108		0.9507 **	0.8448 *	

** *p* = 0.02, * *p* = 0.05.

**Table 8:** Reduction of the aggressiveness of the winter wheat genotypes at different dilutions across isolates and experiments, the measure by DON contamination (mg/kg) 2013–2014. (**A**) Dilution rate, DON mg/kg. (**B**) Correlations between genotype responses to dilution rates.

**(A) Dilution rate, DON mg/kg**												
**Genotypes**	**Normal**		**1:1**		**1:2**		**1:4**	**1:8**		**1:16**		**Mean**
GK Garaboly	22.06		17.23		17.14		19.85	13.22		11.72		16.87
GK Futár	13.37		18.96		14.26		16.55	11.88		7.75		13.79
GK Fény	13.34		12.26		12.43		9.06	8.25		9.71		10.84
F 569/...F.379	11.74		12.30		9.25		10.59	6.70		5.60		9.37
GK 09.09	12.21		10.22		8.88		9.96	7.12		6.77		9.19
GK Csillag	9.10		7.97		9.56		6.33	6.06		4.81		7.30
F 569/..Kő	6.07		3.89		3.45		3.67	1.62		2.25		3.49
Mean	12.56		11.83		10.71		10.86	7.84		6.94		10.12
LSD 5% genotype												1.30
LSD 5% dilution												0.99
**(B) Correlations between genotype responses to dilution rates**												
**Genotypes**		**GK Garaboly**		**GK Futár**		**GK Fény**			**F 569/81.F.379**		**GK 09.09**	
GK Futár		0.6117										
GK Fény		0.5851		0.3432								
F 569/81.F.379		0.8698 *		0.8605 *		0.6633						
GK 09.09		0.9583 ***		0.6203		0.7096			0.9249 **			
GK Csillag		0.6771		0.5110		0.8576 *			0.6865		0.6920	
F 569/Kő		0.9085 **		0.4196		0.7967 *			0.8187 *		0.9633 ***	

*** *p* = 0.001, ** *p* = 0.01. * *p* = 0.05, genotype reactions between the six dilutions (*n* = 6).

**Table 9:** The resistance of wheat genotypes against different *Fusarium* isolates expressed in DI, FDK and DON responses, 2013–2014. (**A**) Disease Index and Isolates %. (**B**) FDK and Isolates %. (**C**) DON and Isolates mg/kg. (**D**) Correlations between traits separately for all isolates.

**(A) Disease Index and Isolates %**						
**Cultivars**	**Fc 52.10**	**Fg 15.38**	**Fc 12375**	**Fg 19.42**	**Mean**	**LSD 5%**
GK Garaboly	26.75	18.00	54.50	17.81	29.26	
GK 09.09	12.79	7.37	29.82	9.65	14.91	
GK Csillag	17.85	11.50	31.23	14.58	18.79	
GK Fény	17.98	10.04	32.07	11.45	17.89	
F 569/81.F.379	11.55	5.68	20.32	8.67	11.55	
F 569/Kő	8.83	3.79	14.45	4.09	7.79	
GK Futár	20.95	11.47	38.32	15.05	21.45	
Mean	16.67	9.69	31.53	11.61	17.38	0.72
LSD 5%					0.96	
	**(B) FDK and Isolates %**					
GK Garaboly	33.91	27.90	77.84	29.49	42.29	
GK 09.09	12.01	7.03	37.83	10.36	16.81	
GK Csillag	16.46	10.58	34.45	15.18	19.17	
GK Fény	17.80	9.47	36.69	12.45	19.10	
F 569/81.F.379	12.11	8.03	30.54	14.26	16.23	
F 569/Kő	8.84	3.93	14.99	4.13	7.97	
GK Futár	20.58	11.78	49.50	15.98	24.46	
Mean	17.39	11.25	40.27	14.55	20.86	1.14
LSD 5%					1.52	
	**(C) DON and Isolates mg/kg**					
GK Garaboly	17.90	8.24	36.05	5.28	16.87	
GK 09.09	17.54	3.23	28.96	5.45	13.79	
GK Csillag	10.71	3.10	26.04	3.51	10.84	
GK Fény	9.38	3.33	19.84	4.91	9.37	
F 569/81.F.379	9.64	2.53	21.83	2.78	9.19	
F 569/Kő	8.43	2.12	16.17	2.48	7.30	
GK Futár	3.89	0.96	7.87	1.24	3.49	
Mean	11.07	3.36	22.40	3.67	10.12	0.85
LSD 5%					1.31	
**(D) Correlations between traits separately for all isolates**						
**Isolates**	**DI/FDK**		**DI/DON**		**FDK/DON**	
Fc 52.10	0.9633 ***		0.8822 *		0.8522 **	
Fg 15.38	0.9295 **		0.8359 **		0.9636 ****	
Fc 12375	0.9759 ***		0.9078 ***		0.9273 ***	
Fg 19.42	0.8758 **		0.6515		0.7398	
Mean for isolate averages	0.9558 ***		0.7827 *		0.9128 ***	

**** *p* = 0.001, *** *p* = 0.01, ** *p* = 0.02, * *p* = 0.05.

**Table 10:** ANOVA of the DI, FDK, and DON data in the inoculum dilution tests, MS values, 2013 and 2014. (**A**) MS values of ANOVA. (**B**) ANOVA between main effects and the two-way interactions.

**(A) MS values of ANOVA**							
**Source of Variance**	**df**	**FHB**	***p***	**FDK**	***p***	**DON**	***p***
Genotype A	6	10513.0	***	24551.9	***	4063.65	***
Dilution B	5	2193.9	***	3897.9	***	1262.95	***
Isolate C	3	36934.9	***	65631.6	***	30111.83	***
Experiment D	2	103459.6	***	64776.6	***	65764.59	***
A × B	30	51.8	***	120.8	***	101.60	***
A × C	18	935.9	***	2066.5	***	741.90	***
A × D	12	488.9	***	1773.5	***	1314.73	***
B × C	15	104.3	***	232.4	***	219.51	***
B × D	10	1130.3	***	886.5	***	808.45	***
C × D	6	6534.4	***	4900.6	***	14240.03	***
A × B × C	90	23.2	n.s.	74.4	***	89.43	***
A × B × D	60	35.7	***	74.6	***	346.19	***
A × C × D	36	149.9	***	339.4	***	100.98	***
B × C × D	30	111.4	***	154.9	***	285.00	***
A × B × C × D	90	37.1	***	114.7	***	86.88	***
Within	1008	26.1		65.0		48.34	
Total	1511						
**(B) ANOVA between main effects and the two-way interactions**							
**Interactions**	**df**	**MS**	***p***	**MS**	***p***	**MS**	***p***
A × B, df–30	A:6	202.95	***	203.24	***	40.00	***
	B:5	42.34	***	32.27	***	12.43	***
A × C, df–18	A:6	11.23	***	11.88	***	5.48	**
	C:3	39.44	***	31.76	***	40.59	***
B × C, df–15	B:5	21.03	***	16.77	***	5.75	**
	C:3	354.11	***	282.41	***	299.60	***

n.s. non-significant, *p* = significance level, *** *p* = 0.001, ** *p* = 0.01, * *p* = 0.05.

**Table 11:** Wheat genotypes investigated at Szeged between 2013–2014 and their abbreviations.

Genotype	Resistance Class	Abbreviation
F 569//Ttj/RC103/3/Várkony/4/Ttj/RC103/3/81.60/NB//Kő	R	F569/Kő
F 569//Ttj/RC 103/3/Várkony/4/Ttj/81.F379	R	F569/81.F.379
GK 09.09	MS	GK 9.09
GK Fény	MR	GK Fény
GK Garaboly	S	GK Garaboly
GK Csillag	MR	GK Csillag
GK Futár	S	GK Futár

R = resistant, MR = moderately resistant, MS = moderately susceptible, S = susceptible.

## Supplementary Materials

The following are available online at https://www.mdpi.com/2223-7747/9/8/943/s1, Figure S1: Petri dish aggressiveness test at different aggressiveness, Table S1: Aggressiveness tests for the isolates used in 2013 on two genotypes differing in seedling resistance.
