# Supplementary material for: The Influence of the Dilution Rate on the Aggressiveness of Inocula and the Expression of Resistance against Fusarium Head Blight in Wheat
Source: Plants (Basel). 2020 Jul 25;9(8):943. doi: 10.3390/plants9080943 (PMC7465623; doi:10.3390/plants9080943)
Supplement: Supplementary file 1 [file plants-09-00943-s001.pdf]

Supplementary materials:

Table S1. Aggressiveness tests for the isolates used in 2013 on two genotypes differing in seedling resistance. 25 seeds were tested in a Petri dish in 5 × 5 binding. The number of healthy germs were counted daily from the 2nd to 6th day. Normal (without dilution) concentration and 1:1, 1:2, and 1:4 dilutions were used.

**Isolate**	**Variety 1 S**	**Days after Sowing / No. of Healthy Germs**					**Mean**	**% to**
		**2**	**3**	**4**	**5**	**6**		**Check**
**Fc 12375/ 2013**	No dilution	0	0	0	0	0	0	0.00
	1:1	0	0	0	0	0	0	0.00
	1:2	0	0	0	0	0	0	0.00
	1:4	0	0	0	0	0	0	0.00
	Check	23	24	24	24	24	23.8	100.00
	Mean % for dilutions						0	0.00

	Variety 2 MS	Days after sowing / No. of healthy germs					Mean	% to
		2	3	4	5	6		check
	No dilution	0	0	0	0	0	0	0.00
	1:1	0	0	0	0	0	0	0.00
	1:2	0	0	0	0	0	0	0.00
	1:4	0	0	0	0	0	0	0.00
	Check	22	23	24	24	24	23.4	100.00
	Mean % for dilutions							0.00

	Variety 1 S	Days after sowing / No. of healthy germs					Mean	% to
		2	3	4	5	6		check
**Fc 52.10 2013**	No dilution	0	0	0	0	0	0	0.00
	1:1	0	0	0	0	0	0	0.00
	1:2	0	0	0	0	0	0	0.00
	1:4	7	8	8	8	8	7.8	32.77
	Check	23	24	24	24	24	23.8	100.00
	Mean % for dilutions							8.19

	Variety 2 MS	Days after sowing / No. of healthy germs					Mean	% to
		2	3	4	5	6		check
	No dilution	0	0	0	0	0	0	0.00
	1:1	0	0	0	0	0	0	0.00
	1:2	1	2	3	3	3	2.4	10.26
	1:4	9	9	8	8	8	8.4	35.90
	Check	22	23	24	24	24	23.4	100.00
	Mean % for dilutions							11.54

	Variety 1 S	Days after sowing / No. of healthy germs					Mean	% to
		2	3	4	5	6		check
**Fg 19.42 2013**	No dilution	2	0	0	0	0	0.4	1.68
	1:1	2	2	1	1	1	1.4	5.88
	1:2	11	7	2	2	2	4.8	20.17
	1:4	14	9	4	4	4	7	29.41
	Check	23	24	24	24	24	23.8	100.00
	Mean % for dilutions							14.29

	Variety 2 MS	Days after sowing / No. Of healthy germs					Mean	% to
		2	3	4	5	6		check
	No dilution	1	0	0	0	0	0.2	0.85
	1:1	4	1	1	1	1	1.6	6.84
	1:2	6	5	3	3	3	4	17.09
	1:4	12	9	6	6	6	7.8	33.33
	Check	22	23	24	24	24	23.4	100.00

	Mean % for dilutions							14.53

	Variety 1 S	Days after sowing / No. of healthy germs					Mean	% to check
		2	3	4	5	6		
**Fg 13.38 2013**	No dilution	0	0	0	0	0	0	0.00
	1:1	3	1	0	0	0	0.8	3.36
	1:2	6	5	3	3	3	4	16.81
	1:4	14	9	8	8	8	9.4	39.50
	Check	23	24	24	24	24	23.8	100.00
	Mean % for dilutions							14.92

	Variety 2 MS	Days after sowing / No.of healthy germs					Mean	% to check
		2	3	4	5	6		
	No dilution	2	1	1	1	1	1.2	5.13
	1:1	3	3	3	2	2	2.6	11.11
	1:2	11	8	6	5	4	6.8	29.06
	1:4	8	6	6	6	3	5.8	24.79
	Check	22	23	24	24	24	23.4	100.00
	Mean % for dilutions							17.52

	Variety 1 S	Days after sowing / No. of healthy germs					Mean	% to check
		2	3	4	5	6		
**Fg 46.06 2013**	No dilution	12	8	8	8	8	8.8	36.97
	1:1	20	18	18	18	18	18.4	77.31
	1:2	21	21	21	21	21	21	88.24
	1:4	19	19	19	19	19	19	79.83
	Check	23	24	24	24	24	23.8	100.00
	Mean % for dilutions							70.59

	Variety 2 MS	Days after sowing / No. of healthy germs					Mean	% to check
		2	3	4	5	6		
	No dilution	15	14	13	13	13	13.6	58.12
	1:1	14	13	13	13	13	13.2	56.41
	1:2	21	18	18	18	18	18.6	79.49
	1:4	23	21	21	21	21	21.4	91.45
	Check	22	23	24	24	24	23.4	100.00
	Mean % for dilutions							71.37

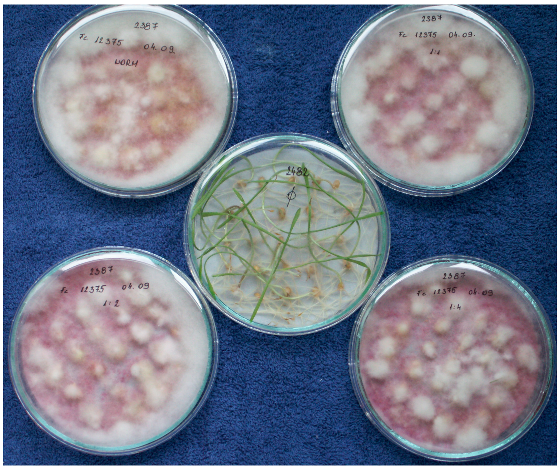

Fc 12375

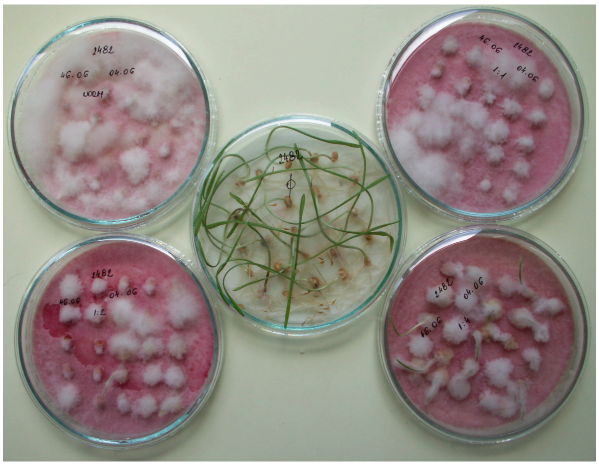

Fg 46.06

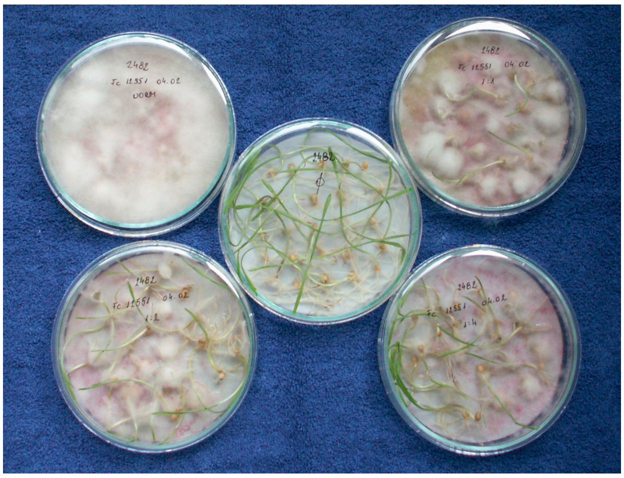

Fc. 12551

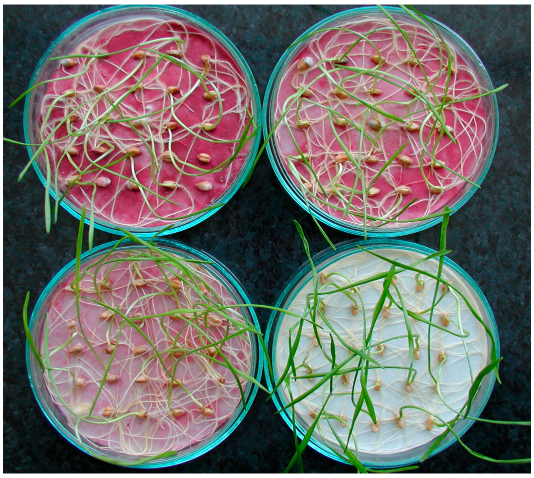

F. avenaceum 17

Figure S1. Petri dish aggressiveness test at different aggressiveness. The pictures are from another year, and demonstrate the aggressiveness differences that are shown by the data in Table S1. Left upper corner: original concentration, right upper: dilution 1:1, lower left: dilution 1:2, low right: dilution 1:4, in the middle non-inoculated check. For F. avenaceum, the 1:8 dilution was absent.
